# Renal tubular acidosis without interstitial nephritis in Sjögren’s syndrome: a case report and review of the literature

**DOI:** 10.1186/s12882-023-03290-3

**Published:** 2023-08-15

**Authors:** Shintaro Hamada, Tomoaki Takata, Kentaro Yamada, Marie Yamamoto, Yukari Mae, Takuji Iyama, Takaaki Sugihara, Miki Takata, Hajime Isomoto

**Affiliations:** 1 Division of Gastroenterology and Nephrology, Tottori University Faculty of Medicine, Yonago, Tottori 683-8504 Japan; 2 Department of Respiratory Medicine and Rheumatology Graduate School of Medicine, Tottori University Hospital, Yonago, Tottori 683-8504 Japan

**Keywords:** Hypokalemia, Renal tubular acidosis, SJÖGREN’S syndrome, H^+^-ATPase, Interstitial nephritis

## Abstract

**Background:**

Renal tubular acidosis is the principal clinical feature associated with tubulointerstitial nephritis in patients with primary Sjögren’s syndrome. Renal tubular dysfunction due to interstitial nephritis has been considered the underlying pathophysiology connecting renal tubular acidosis and primary Sjögren’s syndrome. However, the detailed mechanisms underlying the pathophysiology of renal tubular acidosis in primary Sjögren’s syndrome is not fully understood.

**Case presentation:**

A 30-year-old woman was admitted with complaints of weakness in the extremities. The patient was hospitalized thirteen years earlier for similar issues and was diagnosed with hypokalemic paralysis due to distal renal tubular acidosis with primary Sjögren’s syndrome. This diagnosis was based on a positive Schirmer's test. Besides, anti-Sjögren’s syndrome-related antigen A was also detected. Laboratory tests indicated distal RTA; however, a renal biopsy showed no obvious interstitial nephritis. Laboratory tests conducted during the second admission indicated distal renal tubular acidosis. Therefore, a renal biopsy was performed again, which revealed interstitial nephritis. Histological analysis of acid–base transporters revealed the absence of vacuolar type H^+^-ATPases in the collecting duct. The vacuolar type H^+^-ATPase was also absent in the past renal biopsy, suggesting that the alteration in acid–base transporters is independent of interstitial nephritis.

**Conclusions:**

This case study demonstrates that vacuolar-type H^+^-ATPases are associated with distal renal tubular acidosis, and distal renal tubular acidosis precedes interstitial nephritis in patients with primary Sjögren’s syndrome.

## Background

Primary Sjögren’s syndrome (pSS) is a chronic systemic autoimmune disease characterized by lymphoplasmacytic infiltration of the lacrimal and salivary glands [1]. Renal dysfunction is often observed in pSS [2]. Peritubular and interstitial infiltration by lymphocytes leads to tubulointerstitial nephritis [1], which is associated with tubular dysfunction. Renal tubular acidosis (RTA) caused by renal acid retention or bicarbonate loss is the principal clinical feature associated with tubulointerstitial nephritis in patients with pSS [3, 4]. Distal RTA (dRTA) is the dominant form found in patients with pSS [4]. dRTA is an important physiological condition in pSS, since it is often accompanied by hypokalemia, leading to hypokalemic paralysis [5, 6]. Renal tubular dysfunction due to interstitial nephritis is considered the underlying pathophysiology connecting dRTA and pSS [5, 7]; however, the mechanism of RTA is not yet fully understood. Several kidney specific proteins are associated with renal tubular dysfunction [8, 9], whereas one of the proposed hypotheses of RTA is that dysfunction of the vacuolar-type H^+^-ATPases (V-ATPases) is responsible for the defect in urinary acidification leading to metabolic acidosis [10]. The aim of this study was to highlight the clinical course of renal manifestations in patient with pSS and to investigate the potential association between RTA and V-ATPases in pSS.

Here, we present a case report of a patient with pSS that has dRTA and hypokalemic paralysis. The patient had dRTA in the absence of interstitial infiltration of lymphocytes and displayed defects in V-ATPase in the collecting duct, suggesting a link between V-ATPase and dRTA. Furthermore, we demonstrate that dRTA preceded interstitial nephritis using two renal biopsies conducted at different time points. To the best of our knowledge, this is the first report demonstrating the involvement of V-ATPases with dRTA without interstitial nephritis in pSS.

## Case presentation

**Fig. 1:**
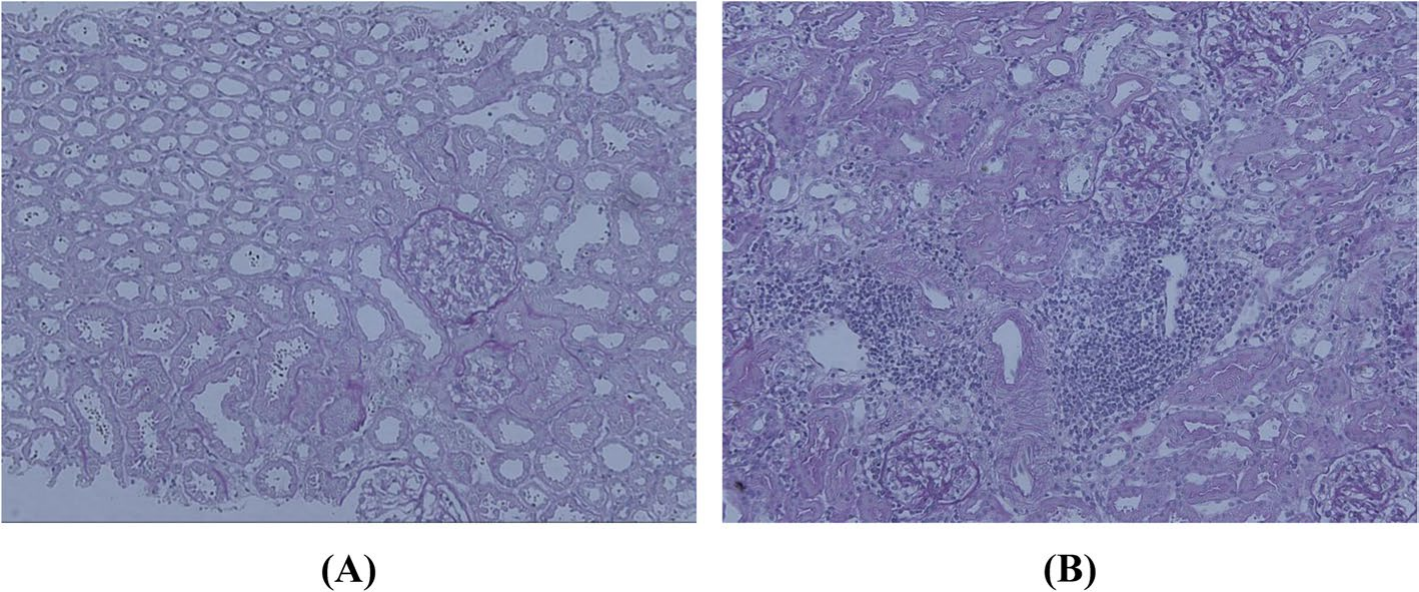
Histological findings of kidney biopsies. Periodic acid-Schiff (PAS) staining of the kidney obtained at initial (**A**) and the present hospitalization (**B**). Interstitial infiltration of lymphocytes was not observed at the initial time whereas it was evident in the biopsied kidney taken during the present hospitalization

A 30-year-old woman was admitted to Tottori University Hospital because of numbness and weakness in her extremities. The patient had a history of hospitalization thirteen years ago with similar complaints. Laboratory examinations during the previous hospitalization revealed hypokalemia with the following readings: 2 mEq/L potassium, 22.6 mmol/L HCO_3_^−^, 20742 μg/L urinary β2-microglobulin, and 28 U/L urinary N-acetylglucosaminidase. In addition, the patient also complained of dry eyes and mouth and tested positive in the Schirmer’s test. Besides, anti-Sjögren’s syndrome-related antigen A was also detected. Renal biopsy taken during the previous hospitalization was negative for interstitial nephritis and glomerular abnormalities (shown in Fig. 1A). During the visit, her symptoms gradually improved, and finally disappeared without any medication.

On the second admission, the patient’s blood pressure was 112/70 mmHg, pulse was 71 beats/min, and body temperature was 36.3 °C. The potassium level was 2.5 mmol/L in whole blood. Plasma aldosterone was 148 pg/mL (range: 29.9–159 pg/mL), and plasma renin activity was 4.3 ng/mL/h (range: 0.3–2.9 ng/mL/h) at rest. Arterial blood samples had a pH of 7.377, pCO_2_ of 32.5 mmHg, serum HCO_3_^−^ of 18.6 mEq/L, and an anion gap of 12.9 mmol/L. Urinary biochemistry showed a urinary β2-microglobulin level of 15,356 μg/L. The urinary pH was 7.5 (> 5.5) and fractional excretion of HCO_3_^−^ was 0.8% (< 3%) despite metabolic acidosis (Table 1). Ultrasonographic and radiological examination of the kidneys revealed no abnormalities. We performed a renal biopsy five days after admission.

**Table 1:** Laboratory data of the patient

WBC	4300	/μL	Free T4	1.06	ng/dL
Neu	64	%	TSH	1.03	μg/dL
Lym	27	%	Renin	4.3	ng/mL
RBC	4.07 × 10^6^	/μL	Aldsterone	148	pg/mL
Hb	10	g/dL	Cortisol	4.66	μg/dL
Hct	31.5	%	Glucose	122	mg/dL
Plt	24.4 × 10^4^	/μL	< Arterial blood >		
TP	7.3	g/dL	pH	7.377	
Alb	3.9	g/dL	pCO_2_	32.5	mmHg
T-Bil	0.5	mg/dL	pO_2_	89.4	mmHg
AST	19	U/L	HCO_3_^−^	18.6	mmol/L
ALT	14	U/L	< Urinalysis >		
ALP	193	U/L	pH	7.5	
Na	139	mmol/L	U. Na	108	mmol/L
K	2.5	mmol/L	U. K	32.1	mmol/L
Cl	108	mmol/L	U. Cr	48.3	mg/dL
Ca	8.7	mg/dL	U. TP/U.Cr	0.33	g/g•Cr
P	3.3	mg/dL	U. NAG	9.8	U/L
Mg	2.1	mg/dL	U. β2MG	15356	μg/L
BUN	14.3	mg/dL	U. HCO_3_^−^	11.7	mmol/L
Cr	0.62	mg/dL	U. Osmolarity	342	mOsm/kg/H_2_O
CRP	0.02	mg/dL			

### Immunofluorescent staining of the kidney

Although the patient had dRTA secondary to pSS, interstitial nephritis was not obvious in the first renal biopsy. To investigate the defect in acid secretion in the absence of interstitial nephritis, we performed immunofluorescence staining for V-ATPase, which has been linked to secretory defects in pSS [3, 11].

Kidney specimens were fixed in formalin and embedded in paraffin. Samples of 2 μm thickness were prepared and deparaffinized, followed by antigen retrieval in citrate buffer (80153, MUTO PURE CHEMICAL, Tokyo, Japan), and then washed with phosphate buffered saline (PBS). After one hour of blocking with a solution containing 3% bovine serum albumin (13–15104, FUJIFILM, Tokyo, Japan), the slides were incubated with primary antibodies at 4 °C overnight. Anti-vacuolar-type ATPase antibody (1:300, sc-55544; Santa Cruz Biotechnology, TX, USA) and anti-phosphorylated aquaporin 2 antibody (1:300, OASG00484; Aviva Systems Biology Corporation, San Diego, USA) were used as primary antibodies. The slides were rinsed with PBS and incubated with fluorescent-conjugated secondary antibodies for one hour at 24 °C, and then mounted in ProLong Gold antifade mounting medium (Invitrogen). Images were acquired using a fluorescent microscope (BZ-X710; Keyence, Tokyo, Japan).

## Results

**Fig. 2:**
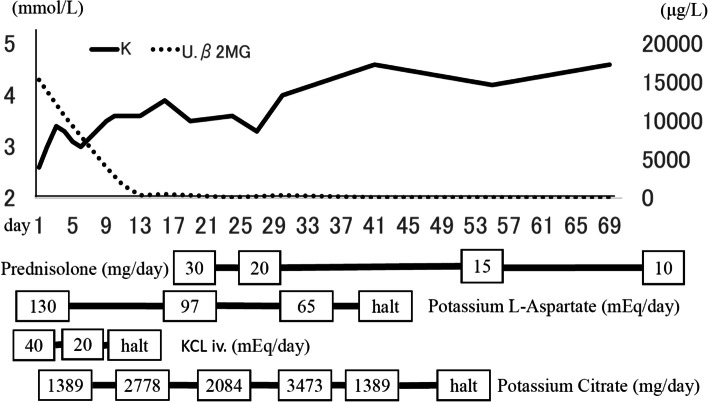
Patient’s clinical course. U.β2MG, urinary β2-microglobulin; KCl iv, intravenous potassium chloride

Histological analysis of the kidney showed interstitial infiltration of mononuclear leukocytes (shown in Fig. 1B). No glomerular abnormalities were observed. The patient was diagnosed with dRTA secondary to pSS. The patient was prescribed oral prednisolone 30 mg/day as well as potassium L-aspartate and potassium citrate supplements. The patient’s symptoms were ameliorated after therapeutic intervention and she was discharged on day 31 (shown in Fig. 2).

**Fig. 3:**
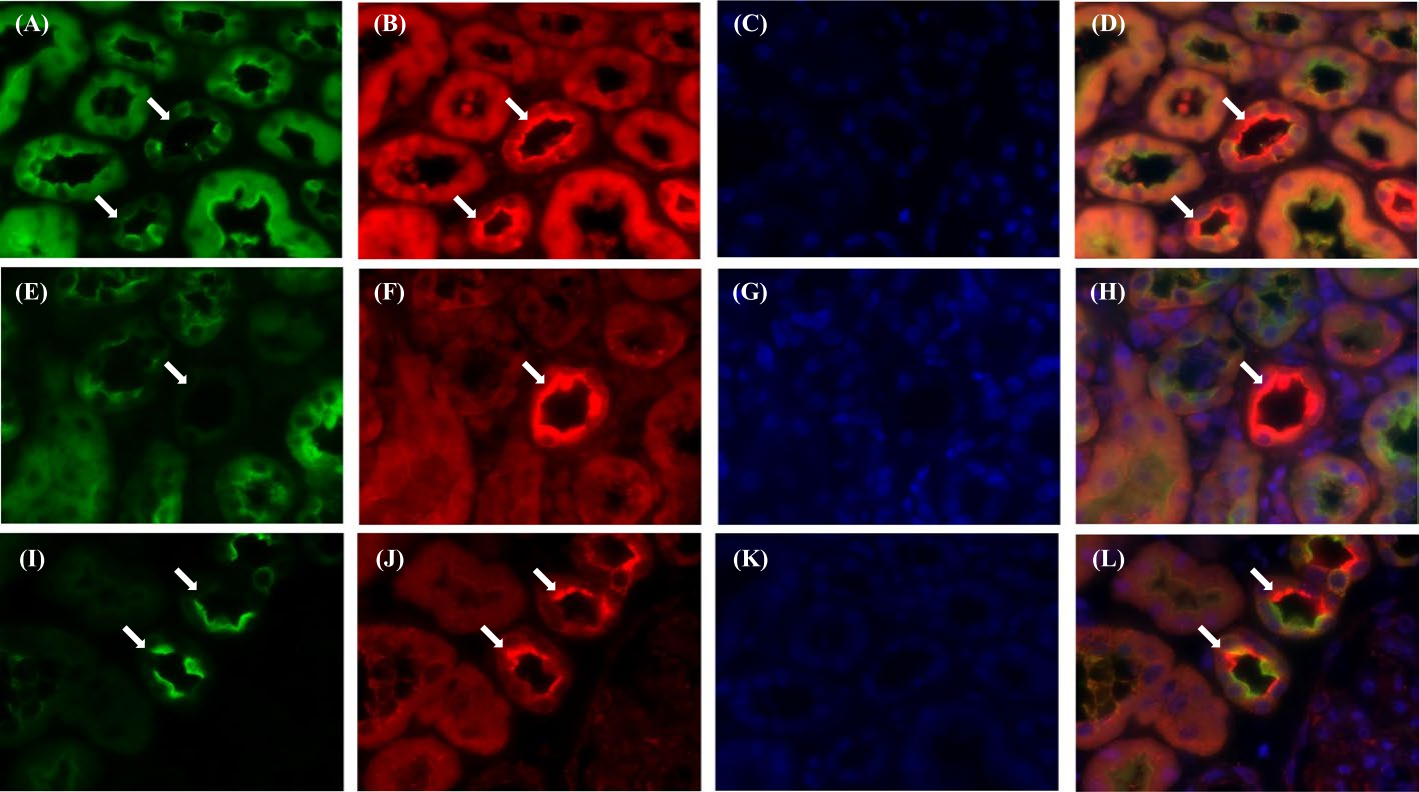
Immunofluorescent staining for vacuolar type H^+^-ATPase. Immunofluorescent staining of the kidney sections obtained from the patient at first (**A** to **D**) and the second (**E** to **H**) biopsy. Kidney sections from a control subject were also showed (**I** to **L**). The vacuolar type H^+^-ATPase signals are absent in the apical membrane of the collecting duct in the present case (**A**, **E**, and **I**), which is indicated by positive phosphorylated aquaporin-2 signals (**B**, **F** and **J**). (**D**), (**H**), and (**L**) represent the merged images. Arrows indicate collecting duct

Immunofluorescent staining of the kidneys revealed that the collecting duct was devoid of V-ATPase signals in the present case (shown in Fig. 3). In particular, the H^+^-ATPase signal was independent of the interstitial infiltration of lymphocytes. These findings suggested that dRTA precedes interstitial nephritis in pSS.

## Discussion/conclusion

In the present case, the patient had dRTA secondary to pSS. Hypokalemia and dRTA occurred despite the absence of interstitial nephritis at the first onset, thirteen years ago. Interstitial nephritis was observed at the second renal biopsy, suggesting that dRTA preceded interstitial nephritis. In addition, V-ATPases were involved in the development of dRTA in pSS, suggesting that The histological assessment of V-ATPase expression may be useful in diagnosing RTA.

Primary SS is a chronic autoimmune inflammatory disease characterized by lacrimal and salivary gland dysfunction. The overall incidence rate of pSS has been estimated at 6.92 per 100,000 person-years [12]. The ratio of women to men has been reported as 27:1 in Asia [13]. Primary SS can involve extraglandular organs, such as the kidney, lung, and vasculature; and sometimes requires immunosuppressive treatment [14]. Previous study in 130 pSS patients showed that sicca syndrome is the major complication in pSS (80% of the patients developed xerostomia and 69.2% developed dry eyes) [6]. Renal involvement is also a major common manifestations of pSS [15, 16] and may overlap with sicca syndrome [6]. The prevalence of renal involvement ranges from 4.9% to 73% [5, 6, 17]. Primary SS can cause glomerular diseases and tubular dysfunction, leading to RTA and Gitelman syndrome [3, 18, 19].

RTA accounts for 73% of pSS cases with renal lesions, with 95.8% of these being dRTA [6]. Although the mechanisms of dRTA in pSS are not clearly understood, the majority of cases are associated with tubulointerstitial nephritis [20]. Previous histological findings have shown that most pSS cases with RTA had interstitial nephritis [17], and several cases of pSS with interstitial nephritis presented with dRTA [21]. However, this case lacked interstitial nephritis despite the existence of dRTA. Although tubulointerstitial nephritis causes tubular dysfunction in pSS, it may not be directly associated with interstitial nephritis. In the present case, we observed improvement of hypokalemia and high urinary β2-microglobulin immediately after the potassium supplement. Deficit in potassium can cause reversible tubular proteinuria, including β2-microglobulin. In fact, low molecular weight proteinuria disappeared following potassium treatment in patients with dRTA [22]. This was further confirmed in rats with hypokalemia [23]. We speculated that hypokalemia caused urinary β2-microglobulin in this case. Although the mechanisms are not fully elucidated, dysfunction of acid–base regulation is associated with the development of dRTA in pSS. Intercalated cells located in the distal convoluted tubules and collecting ducts play an important role in regulating acid balance via the V-ATPases and Cl^−^/HCO_3_^−^ exchangers (anion exchanger 1 and pendrin) [24–26]. Several reports have shown that these transporters, especially V-ATPases, are undetectable in pSS with dRTA [27–30]. In addition, autoantibodies against V-ATPases in intercalated cells exist in the serum of patients with pSS [31, 32]. These findings suggest that V-ATPases in the intercalated cells located in the collecting tubules are associated with the dysfunction of proton secretion in pSS. In the present case, we confirmed that V-ATPases was absent despite no evidence of interstitial nephritis. This finding suggests that autoantibodies directed against intercalated cells are responsible for the development of dRTA. The explanation that dRTA does not directly depend on interstitial nephritis but depends on autoantibody against V-ATPase is compatible with the finding that dRTA precedes interstitial nephritis. Similarly, involvement of lacrimal and salivary glands occurred after the onset of RTA [28].

This study has some limitations. Although lymphocyte infiltration in the interstitium was not evident at the first renal biopsy, sampling bias may have occurred. However, V-ATPases were absent in the area lacking interstitial nephritis. Second, the expression of V-ATPases is not an exclusive cause of dRTA.

Although the role of intercalated cells and the pathophysiology of dRTA should be clarified, we investigated the role of V-ATPases in identifying the unknown cause of dRTA in pSS. In addition, it should be noted that dysfunction of the distal nephron preceded interstitial nephritis in the present case. In conclusion, dRTA can occur without interstitial nephritis in patients with pSS, and V-ATPases in collecting duct cells can be associated with dRTA.

## Data Availability

All data generated or analysed during this study are included in this published article.

## Abbreviations

pSS: Primary Sjögren’s syndrome
RTA: Renal tubular acidosis
dRTA: Distal renal tubular acidosis
V-ATPase: Vacuolar-type H^+^-ATPase
